# Burden of Crohn’s disease in East Asia: a 34-year analysis with projections to 2060

**DOI:** 10.1007/s00384-026-05217-4

**Published:** 2026-08-08

**Authors:** Tianqi Liu, Ruixue Li, Zinan Zhao, Zi Liang, Tian Zhang, Fei Zhao, Yue Wang, Wenfeng Xu, Pengfei Jin

**Affiliations:** 1 Department of Pharmacy, Beijing Hospital, National Center for Gerontology, National Clinical Research Center for Gerontology, The Key Laboratory of Geriatrics of NHC, Institute of Geriatric Medicine, Chinese Academy of Medical Sciences, Beijing, 100730 P.R. China; 2 Department of Pharmacy Administration and Clinical Pharmacy, School of Pharmaceutical Sciences, Peking University, Beijing, 100191 P.R. China; 3 Department of Pharmacy, Xizang Autonomous Region People’s Hospital, Xizang, 850000 P.R. China

**Keywords:** Crohn disease, Prediction, Epidemiological trends, Global burden of diseases

## Abstract

**Purpose:**

Crohn’s disease has transitioned from a rare condition to a significant public health concern in East Asia, yet comparative evidence across countries and long-term projections remain limited. We assess national trends in the burden of Crohn’s disease in China, Japan, and the Republic of Korea from 1990 to 2023 and to estimate future patterns through 2060.

**Methods:**

Data were obtained from the Global Burden of Disease Study 2023. We assessed prevalence, mortality and DALYs, reporting on numbers, rates, age-standardized prevalence rate, age-standardized mortality rate, age-standardized DALYs rate. Decomposition analysis was applied to quantify contributions from population growth, population aging, and shifts in disease epidemiology. Projections for 2024 to 2060 were generated using a Bayesian age–period–cohort model.

**Results:**

All three countries experienced substantial increases in Crohn’s disease burden between 1990 and 2023. Prevalence and DALYs was greatest among males aged 50–54. Projections indicated continued increases in disease burden through 2060. China was projected to retain the largest number of prevalent cases by 2060, while total DALYs in Japan and the Republic of Korea were projected to exceed those in China.

**Conclusion:**

Crohn’s disease burden has risen steadily across East Asia over the past 34 years, with men and middle-aged and older adults disproportionately affected. These findings highlight the need for country-specific prevention and healthcare strategies. China should prioritize early detection and primary care capacity, while Japan and the Republic of Korea require integrated management and long-term care systems to support aging patient populations.

**Supplementary Information:**

The online version contains supplementary material available at https://doi.org/10.1007/s00384-026-05217-4.

## Introduction

Crohn’s disease (CD) is a major type of inflammatory bowel disease, characterized by recurrent inflammation that may involve any part of the gastrointestinal tract from mouth to anus. It leads to progressive intestinal injury, chronic disability, and substantial healthcare and economic burden [1]. Its clinical manifestations are heterogeneous and the disease course is prolonged and relapse-prone. Extraintestinal manifestations occur in approximately 21–47% of patients and worsen long-term outcomes, including increased risks of hospitalization, complications, and surgery [2]. Since the early twenty-first century, the global incidence of CD has increased, with particularly rapid growth in newly industrialized regions [3].

In Asia, rapid socioeconomic development, urbanization, and lifestyle changes have shifted CD from a rare disorder to a common chronic disease [2]. Changes in diet, physical activity, hygiene and sanitation, breastfeeding practices, smoking, and early-life exposure to antibiotics have been implicated as key environmental drivers of the rising incidence of CD in Asia [4–7]. The genetic architecture of CD in Asian populations is less well defined. Several genotype–phenotype associations identified in Western cohorts, such as NOD2 and ATG16L1, are not consistently observed in Asia, although emerging studies suggest the presence of population-specific risk alleles [8].

However, systematic and quantitative estimates of CD burden in Aisa remain limited, and regional heterogeneity is not fully characterized. To address this gap while ensuring comparability across settings, we focused on China, Japan, and the Republic of Korea (ROK), three geographically proximate countries with broadly similar genetic and cultural backgrounds. These countries are high-income or rapidly developing economies undergoing rapid demographic and epidemiological transitions, including population aging and lifestyle changes associated with industrialization. Moreover, they represent settings where the burden of CD has increased markedly in recent decades [9] and where relatively robust and comparable health data systems are available [10], enabling reliable cross-national and temporal analyses. Comparative analysis across these countries can clarify regional patterns in the burden of CD and demonstrate how different stages of socioeconomic development, demographic transition, and health-care system organization influence disease trends.

The Global Burden of Disease (GBD) framework provides standardized estimates of prevalence, mortality, and disability-adjusted life years (DALYs). Previous analyses based on GBD assessed the burden of inflammatory bowel disease [11–13]. The GBD 2023 release provides standalone estimates for CD, enabling robust disease-specific epidemiologic assessment. Using GBD 2023 data, we assessed CD burden in China, Japan, and the ROK from 1990 to 2023. We used decomposition analysis to quantify contributions of population aging, population growth, and epidemiologic change, and applied a Bayesian age–period–cohort (BAPC) model to project trends through 2060. This study aims to characterize the temporal patterns of CD burden in East Asian countries and to provide evidence to guide prevention and management strategies in the region.

## Methods

### Data sources

The Global Burden of Disease (GBD) database is a standardized international platform that compiles and harmonizes epidemiological data on diseases, injuries, risk factors, and health outcomes. GBD 2023 presents the latest epidemiological insights into 375 diseases and injuries across 204 countries and regions from 1990 to 2023. For this analysis, CD prevalence, mortality, DALYs, age-standardized prevalence rate (ASPR), age-standardized mortality rate (ASMR), age-standardized DALYs rate (ASDR) from 1990 to 2023 were obtained from the Global Health Data Exchange (GHDx) query tool (https://ghdx.healthdata.org/) to assess temporal trends and epidemiological patterns. DALYs quantify population-level disease burden by combining years of life lost due to premature mortality with years lived with disability.

### Statistical analysis

#### Decomposition analysis

We implemented a decomposition approach pioneered by Das Gupta to quantify the contributions of multiple drivers to temporal changes in the burden of Crohn’s disease, including population growth, population aging, and epidemiological change [14]. Population growth reflects changes in total population size, population aging represents shifts in age structure, and epidemiological change captures variations in age-specific rates, independent of demographic factors. The decomposition analysis quantified each factor’s contribution to the overall temporal trend and identified whether it increased or reduced the burden of CD. Detailed calculation procedures and formulas for the decomposition analysis are provided in the Supplementary Methods.

#### Joinpoint analysis

We applied Joinpoint regression to the ASPR, ASMR, and ASDR of CD to identify statistically significant change points in temporal trends over the study period. This approach allowed us to delineate intervals of significant increase or decrease in disease burden. Model parameter optimization was performed through permutation testing employing a significance level (α) set at 0.05 and 4,499 permutations. The allowable number of joinpoints was constrained to 0–6, and the software automatically selected the optimal model. Trend inflection points were identified using a segmented log-linear regression model. Detailed model equations and parameter definitions are described in the Supplementary Methods. All analyses were conducted using Joinpoint software (version 5.3.0.0).

#### BAPC model projection

The BAPC framework provides a robust methodology for characterizing long-term trends in chronic diseases and projecting future disease burden. It accommodates large parameter spaces and sparse population data while maintaining numerical stability and reliable estimation. Statistical inference is implemented using Integrated Nested Laplace Approximations (INLA), which enables efficient computation of marginal posterior estimates and, when forecasting is of interest, predictive distributions that naturally account for Poisson variability. This approach generates both age-specific and age-standardized projections and mitigates many convergence issues commonly encountered with Markov chain Monte Carlo (MCMC) methods. In this study, we applied the “BAPC” R package to project Crohn’s disease (CD) burdens from 2024 to 2060, based on GBD 2023 estimates and demographic projections from the IHME. Additionally, a sensitivity analysis was performed using alternative population projections from the United Nations World Population Prospects (UN WPP) to evaluate the robustness of the long-term projections. All analyses were performed in R (version 4.4.3). A detailed description of the Bayesian modeling procedures, including prior specifications and model fitting details, is provided in the Supplementary Methods and Supplementary Table S2.

## Results

### Burden of CD in China, Japan, and the ROK

China had the highest CD burden among the three countries and exhibited a pronounced upward trend over time. Prevalent cases increased from 21,575 (95% UI, 16,534–28,106) in 1990 to 62,291 (95% UI, 49,900–77,787) in 2023, while ASPR rose from 1.86 (95% UI, 1.45–2.39) to 3.56 per 100,000 (95% UI, 2.79–4.49). Japan and the ROK showed similar increases in both prevalence counts and ASPR. Joinpoint regression identified six significant joinpoints for China (1995, 2001, 2006, 2010, 2016, 2019), five for Japan (1992, 1995, 2000, 2005, 2010), and four for the ROK (2000, 2007, 2010, 2016). In China, DALYs attributable to CD increased from 11,038 (95% UI, 6,891–16,974) in 1990 to 16,296 (95% UI, 11,725–21,485) in 2023, whereas ASDR decreased slightly from 0.99 (95% UI, 0.62–1.53) to 0.95 per 100,000 (95% UI, 0.68–1.24). Joinpoint analysis further identified significant change-points in ASDR trend in 1995, 2001, 2006, 2010, 2016, and 2019. Japan and the ROK similarly demonstrated increasing DALYs alongside declining ASDR. Joinpoint regression detected significant joinpoints for Japan in 1994, 2001, 2005, 2010, and 2014, and for the ROK in 2001, 2010, and 2016. Mortality of CD remained low across all three countries. In China, deaths increased slightly from 166 (95% UI, 79–298) in 1990 to 207 (95% UI, 121–317) in 2023, while ASMR declined overall, with a clear downward shift after 2005. ASMR decreased steadily in the ROK, whereas Japan showed a decline until 2017, followed by a modest subsequent increase (Supplement Figures S1–S3; Table 1).

**Table 1:** The burden of CD in 1990 to 2023 in China, Japan and Republic of Korea

Year	Location	Sex	Number (95% UI)			Age-standardized rate per 100,000 population (95% UI)		
			Prevalence cases	Deaths	DALYs	Prevalence rate	Mortality rate	DALY rate
1990	China	Both	21575 (16534,28106)	166 (79,298)	11038 (6891,16974)	1.86 (1.45,2.39)	0.02 (0.01,0.04)	0.99 (0.62,1.53)
		Male	9952 (7497,12910)	71 (25,150)	5099 (2870,8898)	1.67 (1.29,2.12)	0.01 (0.01,0.03)	0.87 (0.49,1.5)
		Female	11622 (9036,15159)	95 (34,221)	5939 (3371,11060)	2.06 (1.6,2.63)	0.02 (0.01,0.05)	1.11 (0.62,2.09)
	Japan	Both	7500 (5939,9239)	79 (61,101)	3496 (2771,4306)	5.19 (4.05,6.51)	0.05 (0.04,0.07)	2.44 (1.94,3.02)
		Male	3390 (2664,4228)	40 (28,58)	1888 (1430,2538)	4.84 (3.77,6.07)	0.06 (0.04,0.09)	2.79 (2.11,3.78)
		Female	4110 (3288,5094)	38 (25,56)	1608 (1231,2045)	5.52 (4.32,6.91)	0.04 (0.03,0.06)	2.11 (1.6,2.68)
	Republic of Korea	Both	7963 (6207,10105)	25 (12,45)	2434 (1631,3351)	17.14 (13.39,21.54)	0.07 (0.03,0.14)	5.38 (3.66,7.54)
		Male	4658 (3631,5933)	12 (5,25)	1336 (866,1988)	20.06 (15.79,25.41)	0.06 (0.03,0.13)	5.81 (3.82,8.56)
		Female	3305 (2530,4343)	13 (4,30)	1099 (665,1808)	14.3 (10.95,18.65)	0.07 (0.02,0.17)	4.89 (2.97,8.14)
2023	China	Both	62291 (49900,77787)	207 (121,317)	16296 (11725,21485)	3.56 (2.79,4.49)	0.01 (0.01,0.02)	0.95 (0.68,1.24)
		Male	28412 (22564,35648)	134 (62,226)	8803 (6143,12193)	3.21 (2.52,4.05)	0.01 (0.01,0.02)	1.02 (0.71,1.43)
		Female	33880 (27340,41877)	72 (30,140)	7493 (5385,10268)	3.92 (3.1,4.94)	0.01 (0,0.01)	0.86 (0.61,1.18)
	Japan	Both	11577 (9448,14168)	103 (79,138)	4103 (3175,5329)	6.61 (5.24,8.28)	0.03 (0.03,0.04)	2.17 (1.7,2.81)
		Male	5125 (4187,6331)	70 (49,99)	2473 (1915,3208)	6.14 (4.88,7.75)	0.05 (0.04,0.07)	2.74 (2.15,3.5)
		Female	6452 (5249,7826)	33 (20,51)	1631 (1173,2296)	7.07 (5.59,8.81)	0.01 (0.01,0.02)	1.62 (1.18,2.27)
	Republic of Korea	Both	11577 (9448,14168)	23 (13,34)	2973 (2052,4129)	21.9 (17.35,27.9)	0.03 (0.02,0.04)	4.41 (2.96,6.04)
		Male	8604 (6979,11057)	13 (7,22)	1773 (1194,2443)	25.46 (20.32,33)	0.04 (0.02,0.06)	5.26 (3.46,7.28)
		Female	6177 (4936,7777)	9 (4,17)	1201 (788,1751)	18.14 (14.08,22.72)	0.02 (0.01,0.03)	3.51 (2.34,5)

*CD* Crohn’s Disease, *DALYs* Disability-adjusted life years

### Decomposition analysis of CD burden in China, Japan, and the ROK

**Fig. 1:**
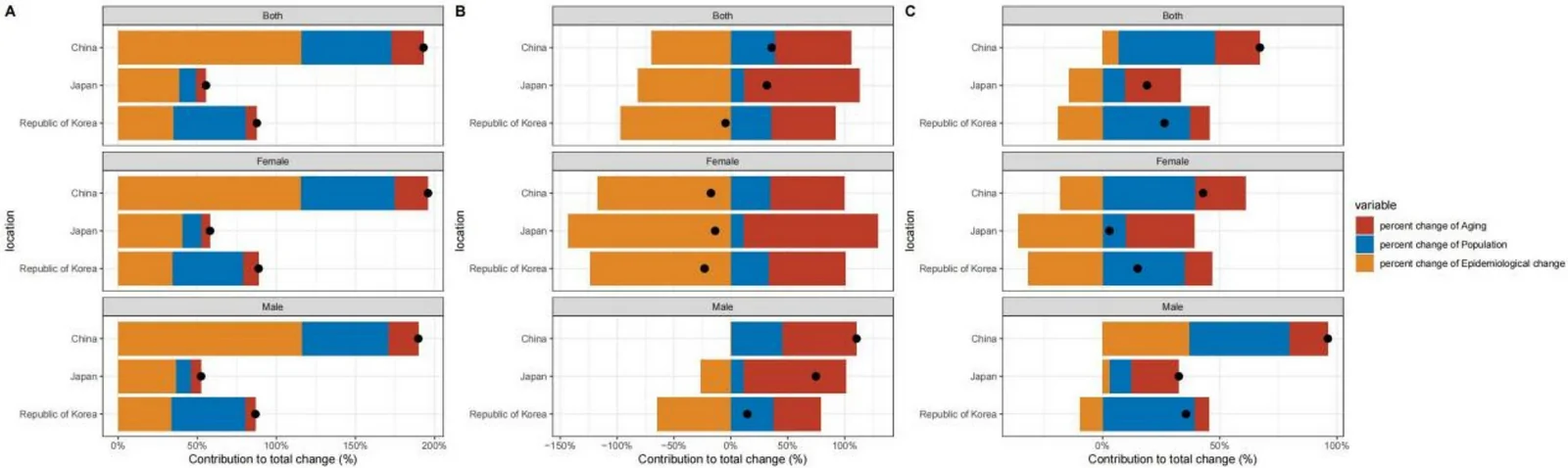
Changes in CD prevalence, mortality and DALYs according to population-level determinants of aging, population growth, and epidemiological change from 1990 to 2023. CD: Crohn’s Disease; DALYs: disability-adjusted life years

Decomposition analysis quantified the contributions of population growth, population aging, and epidemiological change to shifts in CD burden from 1990 to 2023 (Fig. 1; Supplementary Table S1). In China, prevalent cases increased by 193.01%, of which 20.32% was attributable to population aging, 57.06% to population growth, and 115.63% to epidemiological change. DALYs increased by 67.14%, and the contributions of aging, population growth, and epidemiological change were 19.27%, 40.99%, and 6.89%. Mortality increased by 55.88%, with corresponding contributions of 67.08%, 38.74%, and − 69.94%.

In Japan, prevalent cases rose by 55.51%, driven primarily by epidemiological change (38.44%), with smaller contributions from population aging (10.72%) and population growth (6.35%). DALYs increased by 18.94%, with contributions of 23.7% from aging, 9.56% from population growth, and − 14.32% from epidemiological change. Mortality increased by 31.64%, and the contributions of population aging, growth, epidemiological change were 102.24%, 11.13%, and − 81.72%. In the ROK, prevalent cases increased by 87.62%, driven by aging (7.15%), population growth (45.47%), and epidemiological change (35.01%). DALYs rose by 26.41%, with contributions of 8.3% and 37.26% from population aging and growth, and − 19.14% from epidemiological change. Mortality declined by − 4.74%, with population aging and growth contributing 56.76% and 35.35%, whereas epidemiological change contributed − 96.85%.

### Burden of CD by sex and age

**Fig. 2:**
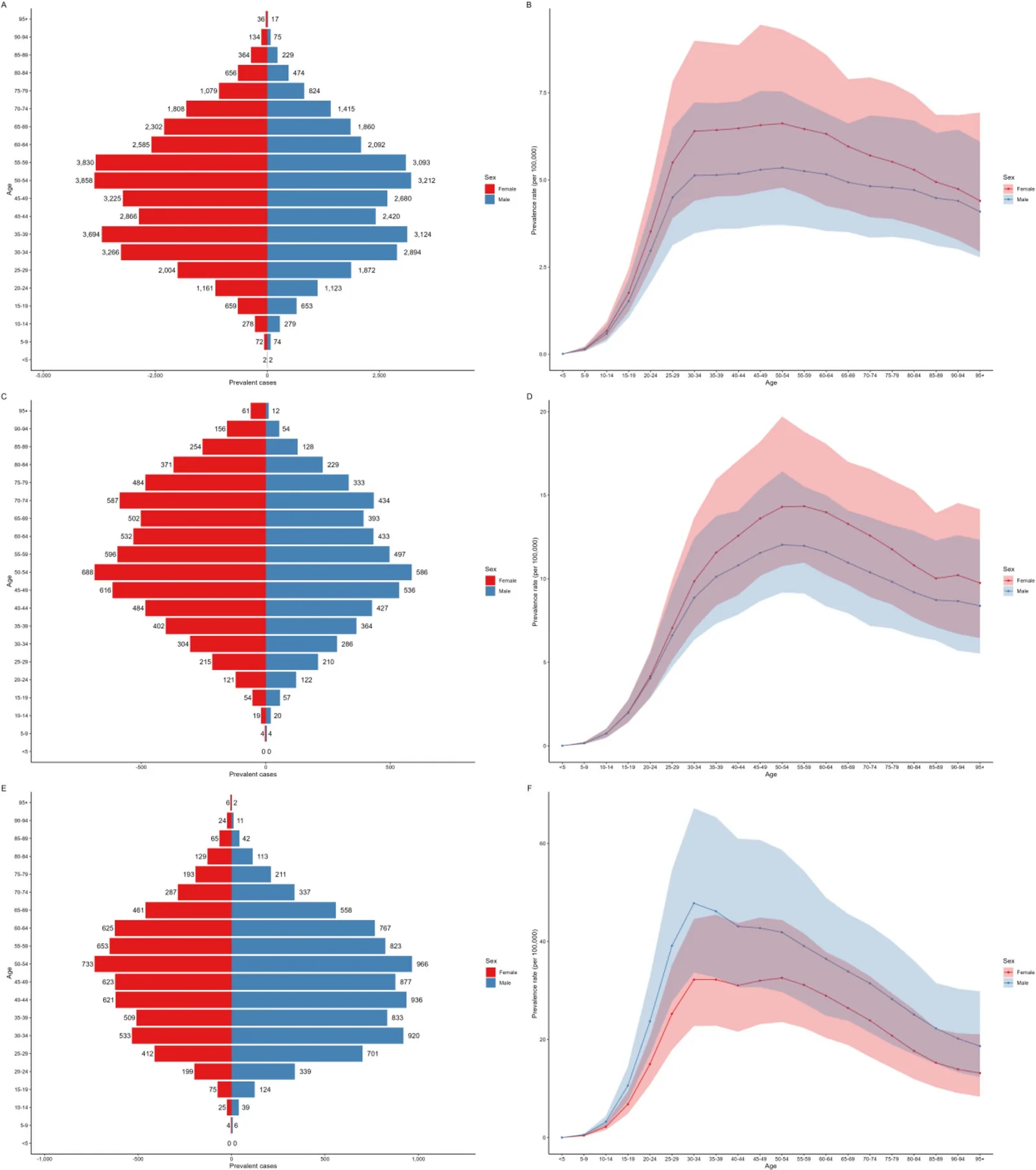
Age-specific numbers and rates (per 100,000 population) of prevalent cases due to CD by gender in 2023. (**A**) China; (**B**) Japan; (**C**) Republic of Korea. CD: Crohn’s Disease; DALYs: disability-adjusted life years

**Fig. 3:**
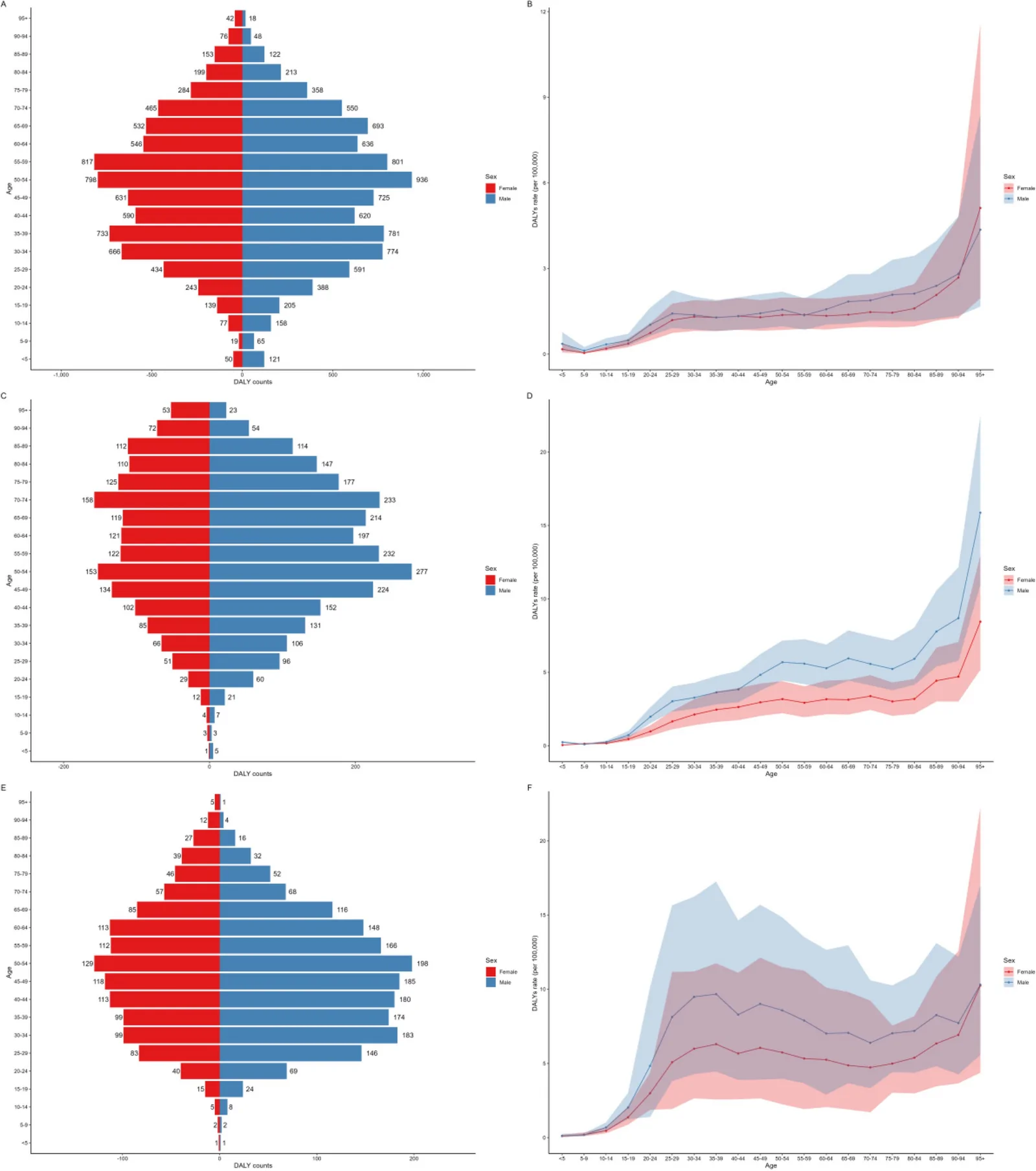
Age-specific numbers and rates (per 100,000 population) of DALYs due to CD by gender in 2023. (**A**) China; (**B**) Japan; (**C**) Republic of Korea. CD: Crohn’s Disease; DALYs: disability-adjusted life years

The highest number of prevalent cases occurred at ages 50–54 in all three East Asian countries. In China, the peak reached 7,070 cases (3,858 females and 3,212 males). In Japan, the corresponding peak was 1,274 cases (688 females and 586 males), and in the Republic of Korea it was 1,699 cases (733 females and 966 males). ASPR peaked at ages 45–49 in China, 50–54 in Japan, and 30–34 in the ROK. DALYs in 2023 were similarly concentrated in middle-aged and older adults. The peak was at ages 50–54 in China (females: 798; males: 936), 55–59 in Japan (females: 153; males: 277), and 50–54 in the ROK (females: 129; males: 198). ASDR increased gradually with age in China and Japan, with sharper rises after age 75, whereas in the ROK ASDR peaked at ages 30–39, and rose again after age 85. Across all countries, deaths and ASMR remained low, increased with age, and peaked after age 75 (Figs. 2, 3, Supplementary Figures S4).

**Fig. 4:**
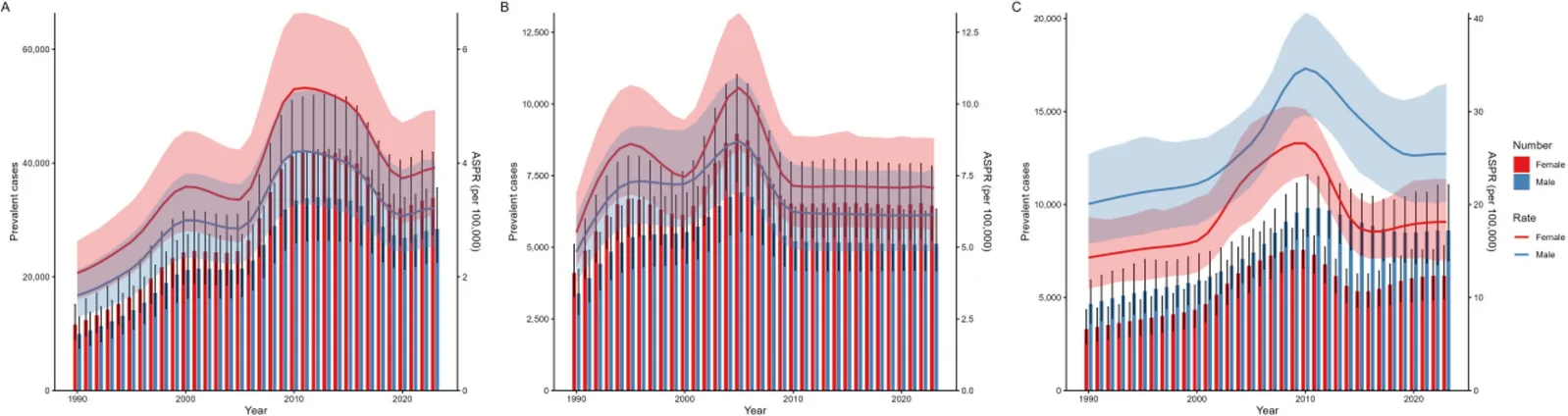
Temporal trends in the number and age-standardized rates (per 100,000 population) of prevalence for CD by sex from 1990 to 2023. (**A**) China; (**B**) Japan; (**C**) Republic of Korea. CD: Crohn’s Disease; ASPR: age-standardized prevalence rate

**Fig. 5:**
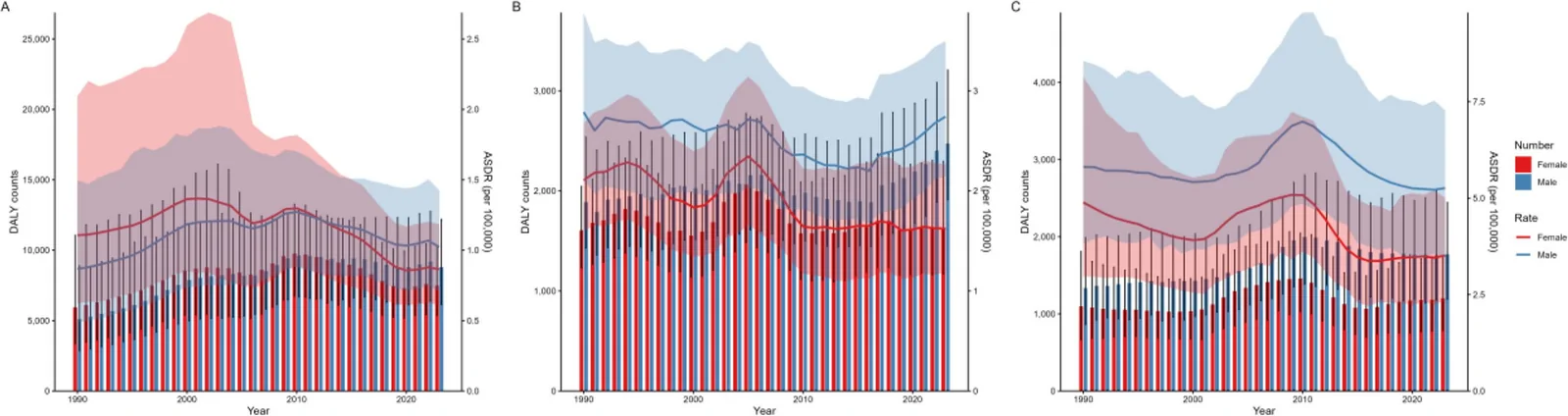
Temporal trends in the number and age-standardized rates (per 100,000 population) of DALYs for CD by sex from 1990 to 2023. (**A**) China; (**B**) Japan; (**C**) Republic of Korea. CD: Crohn’s Disease; ASDR: age-standardized disability-adjusted life years rate; DALYs: disability-adjusted life years

**Fig. 6:**
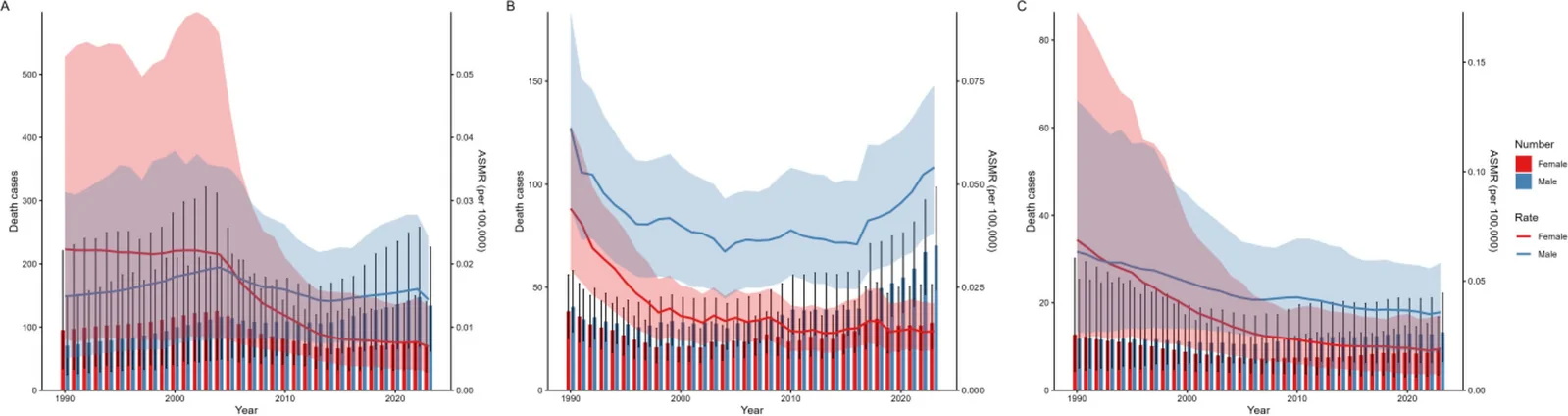
Temporal trends in the number and age-standardized rates (per 100,000 population) of mortality (C) for CD by sex from 1990 to 2023. (**A**) China; (**B**) Japan; (**C**) Republic of Korea. CD: Crohn’s Disease; ASMR: age-standardized mortality rate

From 1990 to 2023, prevalent cases and ASPR increased in all three countries, with the fastest growth in China. DALYs, ASDR, mortality and ASMR mortality remained stable or declined slightly. Persistent sex differences were observed in CD burden. In the ROK, males consistently exceeded females in prevalence, DALYs, mortality cases, ASPR, ASDR, and ASMR. In China and Japan, males bore a higher burden and experienced larger increases in DALYs, ASDR, ASMR, and mortality than females, while females had a higher prevalence cases and ASPR (Figs. 4, 5, 6).

### BAPC modeling of thyroid disease trends in China, Japan, and the Republic of Korea

**Fig. 7:**
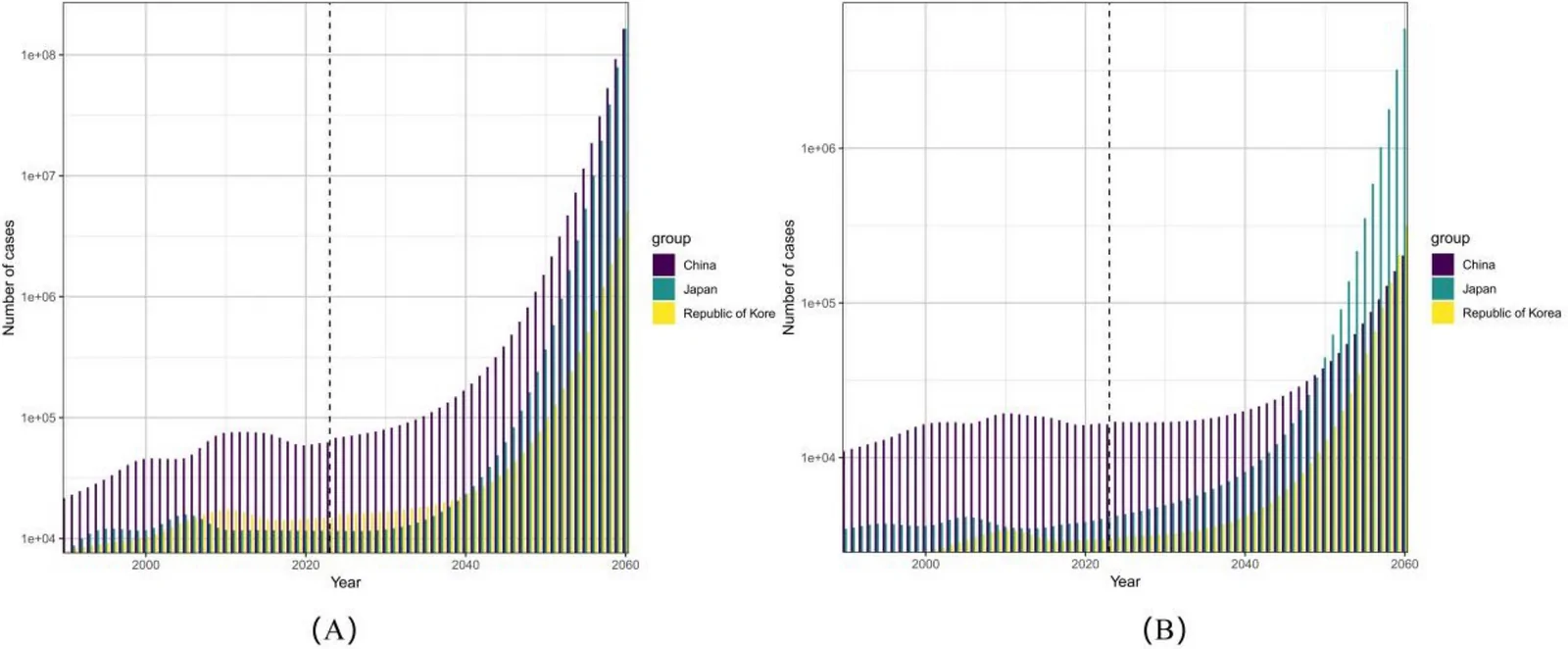
The projections of prevalence cases (**A**) and DALYs (**B**) with CD from 2024 to 2060 in China, Japan and Republic of Korea. CD: Crohn’s Disease

Using the BAPC model, we projected the CD burden in China, Japan, and the ROK for 2024–2060. Prevalent cases and DALY counts were projected to rise markedly in all three countries. By 2060, China was projected to continue to have the largest number of prevalent cases—exceeding 1 × 10^8—while Japan showed the greatest relative increase. For DALYs, the rise in China was slower than in Japan and the ROK. Consequently, by 2060 the DALY counts in Japan and the ROK were projected to surpass those in China, exceeding 1 × 10^6 and 1 × 10^5 (Fig. 7). Furthermore, sensitivity analyses using alternative population inputs from the UN WPP demonstrated consistent long-term trends, supporting the robustness of our projection findings (Supplementary Table 3).

## Discussion

This study provide a comprehensive analysis of the CD burden in China, Japan and the ROK with GBD 2023 data. From 1990 to 2023, prevalent cases, deaths, DALYs, and ASDR increased across all three countries, whereas ASMR and ASDR declined slightly. Prevalence and DALYs was greatest among males aged 50–54. Projections based on a BAPC model suggested continued increases through 2060 in all three countries. These findings necessitate targeted strategies focusing on lifestyle modification, dietary interventions, and enhanced healthcare access at the national level.

Our findings are consistent with prior evidence indicating a sustained rise in CD incidence across East Asia, particularly in China. CD was most common in high-income Western regions, such as North America, Europe, and Oceania. However, the twenty-first century has witnessed a shift [1], with incidence plateauing in high-income countries and increasing in previously low-incidence regions such as Asia and South America. This transition potentially was associated with changes in lifestyle, diet, and pollution exposure [3, 15]. In the ROK, a population-based inception cohort reported a steady rise in incidence of CD from 0 in 1986 to 2.42 per 100,000 in 2015 [16]. Similar upward trends have been documented in Japan, where annual incidence increased from 0.002 per 100,000 in 1955 to 2.0 per 100,000 during 1986–2014 [17–19]. In mainland China, a meta-analysis indicated that national incidence of CD rose from 0.28 per 100,000 during 1950–2002 to 0.85 per 100,000 during 1950–2007 [20]. Notably, the apparent increase in age-standardized rates in the post-COVID period may partly reflect changes in healthcare-seeking behavior and diagnostic practices rather than a true epidemiological surge. Disruptions in healthcare access during the pandemic may have led to delayed diagnoses, followed by a rebound in case detection as services resumed [21, 22].

Previous research indicated that risk factors for CD involve both genetic and environmental influences. Family history is the strongest genetic risk factor, but environmental factors also play a significant role [23]. Studies of migrants showed that moving from low- to high-incidence regions increases CD risk, with an even higher risk observed in their offspring [1]. Since the early twenty-first century, rapid urbanization and industrialization in East Asia have coincided with rising CD incidence, likely driven by lifestyle changes such as Westernized diets, smoking (including passive exposure), antibiotic usage, and reduced physical activity [2]. Dietary patterns have shifted from high-fiber, low-fat foods to diets rich in sugar, omega-6 fatty acids, polyunsaturated fats, total fats, and processed foods containing additives, which are associated with increased CD risk [24]. Reduced dietary fiber intake, especially when alternating between high- and low-fiber foods, leads to decreased gut microbial diversity, further contributing to disease development [3, 25, 26]. Epidemiological evidence suggested that early-life exposures—such as breastfeeding and green space contact—may lower disease risk, while childhood antibiotic use appears to increase it [26, 27]. Additionally, use of oral contraceptives, aspirin, and nonsteroidal anti-inflammatory drugs has been linked to higher CD risk [2]. Fast-food consumption in the ROK increased 4.6-fold, and animal-protein intake in China tripled, paralleling the sharp rise in CD prevalence [28–30]. These patterns provide epidemiological support for the “diet–microbiota–immune regulation” hypothesis.

Many studies had not identified a sex difference in CD prevalence in Western countries, while males consistently dominated prevalent cases across Asia–Pacific regions [31–33]. The male-to-female ratio for adjusted CD incidence were 3.3:1 in the ROK, 2:1 in Japan, and 1.15:1 in mainland China [34]. Our analysis found that in China and Japan, males had lower prevalence but greater DALYs and mortality than women. In the ROK, males exceeded females in all three metrics. These results may reflect higher rates of smoking, alcohol use, and occupational stress among men during East Asia's rapid industrialization. Moreover, men typically exhibit delayed care-seeking and lower adherence to health management, potentially leading to more severe disease at diagnosis—helping explain why male DALYs and deaths increased more rapidly despite lower case counts.

Decomposition analysis of the CD burden from 1990 to 2023 indicated that the increase in DALYs and mortality in China, Japan, and ROK was primarily driven by population growth and aging, while epidemiological changes partially offset this upward trend. The analysis also identified epidemiological changes as one of the major contributor to rising prevalence in three countries. These findings suggested that improvements in disease awareness, healthcare capacity, diagnostic practices, and access to advanced therapies have enabled earlier case identification and reductions in disease severity and mortality risk. In the Republic of Korea, the incorporation of colonoscopy into the national cancer screening program, and timely use of biologics combined with structured monitoring contributed to declining mortality [35, 36]. China introduced biologics such as infliximab in 2006 and subsequently incorporated them into national insurance coverage [36]. However, rapid urbanization continues to drive increases in prevalence, creating dual challenges of expanding treatment coverage and managing a growing patient population.

CD most commonly occurs in individuals between 20 and 35 years of age, and a smaller second peak around 50 years has also been described [37]. Because CD is typically diagnosed at a young age and is associated with low mortality, its prevalence is expected to continue increasing, and middle-aged and older patients are becoming a rapidly expanding group [38]. Our results showed prevalent cases and DALYs were concentrated in 30–59 years, peaking at 50–54 years in China and the ROK. In Japan, prevalent cases and DALYs were concentrated at 45–74 years. Prevalent cases peaking at 50–54 years and DALYs peaking at 55–59, reflecting Japanese older population structure. The concentration of disease burden among working-age adults implied substantial socioeconomic and productivity losses. Moreover, studies had shown that CD in children younger than 10 years is increasingly common [3], suggesting that the overall burden may shift to earlier ages and continue to intensify.

BAPC model projections to 2060 indicated substantial increases in both prevalent cases and DALYs across all three countries. Given China's large population, it was expected to retain the highest prevalent cases but its relative growth was comparatively modest. Japan and the ROK were projected to exceed China in DALYs. These results reflect differences in aging patterns and socioeconomic stages across the three countries. In China, rapid population aging has markedly expanded the size of the elderly population [39]. Long-term exposure to risk factors—including high-fat/high-salt diets, antibiotic overuse, and urban environmental pollution—may further compromise gut microbiota and immune function, increasing vulnerability to disease in older adults. At the same time, improvements in health management have enhanced case detection in this age group. Japan and the ROK have achieved effective disease control through well-developed healthcare systems [40, 41]. However, the expanding population aged ≥ 60 years has paradoxically increased absolute DALYs, reflecting prolonged survival and higher CD prevalence in aging populations.

Based on these observations, stratified prevention and control strategies are warranted. In China, it is necessary to strengthen early identification and build primary-care capacity to meet the growing demand for services. Japan and Korea should strengthen elderly care systems. Evidence indicates that early investment in effective prevention programs can reduce chronic disease incidence and complications, thereby conserving healthcare and social resources [42]. Consolidating advances in early detection, early treatment, and sustained long-term management remains warranted. Modifiable factors—including smoking cessation, judicious antibiotic use, promotion of breastfeeding, and provision of appropriate dietary counseling—may reduce CD incidence and improve long-term outcomes.

This study had several limitations. First, although the GBD database provides reliable data, the absence of individual-level information on disease burden constrained our capacity to conduct more granular and personalized analyses. Second, the scarcity of subnational data in most countries limited the applicability to domestic policymaking, particularly at provincial or regional levels. Third, although age- and sex-specific patterns were examined, other potential modifiers—such as socioeconomic status, lifestyle, and comorbidities—could not be explored due to data constraints. Finally, although projections of future trends are valuable, the relatively long projection horizon compared with the observed data period may introduce additional uncertainty. While the BAPC model incorporates uncertainty through its probabilistic framework, the width of uncertainty intervals increases over time, and projections may be sensitive to underlying model assumptions. To evaluate this potential risk, we performed a sensitivity analysis, which demonstrated consistent long-term trends and supported the overall robustness of our findings. Nevertheless, such models cannot fully account for sudden future shifts in healthcare systems, policy interventions, or emerging therapies, which may substantially alter disease trajectories. Future efforts should focus on improving data quality through multicenter registry studies and elucidating environment–gene interactions to refine prevention and control strategies.

## Conclusion

This study comprehensively evaluated the burden of CD across China, Japan, and the ROK over the past three decades. The burden of CD has risen substantially across three counties, with men and middle-aged and older adults disproportionately affected. China is currently experiencing a phase of rapid growth in CD prevalence, and should strengthen early identification and build primary-care capacity. Japan and the ROK face challenges driven by population aging and would benefit from strengthening geriatric care systems. These findings offer a valuable foundation for targeted CD prevention and control strategies in East Asia. Through comprehensive and collaborative efforts, the growing burden of CD can be effectively mitigated, leading to better patient prognosis and quality of life.

## Supplementary Information

Below is the link to the electronic supplementary material.

Supplementary file1 (DOCX 928 KB)

## Data Availability

The data that support the findings of this study are available in the 2023 Global Burden of Disease Study at https://ghdx.healthdata.org/gbd-2023. These data were derived from the following resources available in the public domain: Global Health Data Exchange (GHDx), https://ghdx.healthdata.org/. Any additional information required to reanalyze the data reported in this paper are available from the corresponding author upon request.
