# Supplementary material for: Burden of Crohn’s disease in East Asia: a 34-year analysis with projections to 2060
Source: Int J Colorectal Dis. 2026 Aug 8;41:176. doi: 10.1007/s00384-026-05217-4 (PMC13736409; doi:10.1007/s00384-026-05217-4)
Supplement: Supplementary file 1 [file 384_2026_5217_MOESM1_ESM.docx]

**Title : Burden of Crohn’s Disease in East Asia: A 34-Year Analysis with Projections to 2060**

*[International Journal of Colorectal Disease](https://link.springer.com/journal/384)*

**Author and institutional affiliations :**

Tianqi Liu^ab*^, Ruixue Li^abc*^, Zinan Zhao^b^, Zi Liang^ab^,Tian Zhang^b^, Fei Zhao^b^, Yue Wang^b^, Wenfeng Xu^b#^, Pengfei Jin^b#^

*Ruixue Li and Tianqi Liu have contributed equally to this work.

Affiliations:

a. Department of Pharmacy Administration and Clinical Pharmacy School of Pharmaceutical Sciences, Peking University, Beijing 100191, P.R. China.
b. Department of Pharmacy, Beijing Hospital; National Center of Gerontology; Institute of Geriatric Medicine, Chinese Academy of Medical Sciences; Beijing Key Laboratory of Assessment of Clinical Drugs Risk and Individual Application (Beijing Hospital), Beijing 100730, P.R. China;
c. Department of Pharmacy, Xizang Autonomous Region People's Hospital, Xizang 850000, P.R. China;

**# Corresponding author:** Pengfei Jin and Wenfeng Xu

Pengfei Jin

Department of Pharmacy, Beijing Hospital; National Center of Gerontology; Institute of Geriatric Medicine, Chinese Academy of Medical Sciences; Beijing Key Laboratory of Assessment of Clinical Drugs Risk and Individual Application (Beijing Hospital), Beijing 100730, P.R. China

E-mail address: j790101@163.com

Tel: +86-13811300092

Wenfeng Xu

Department of Pharmacy, Beijing Hospital; National Center of Gerontology; Institute of Geriatric Medicine, Chinese Academy of Medical Sciences; Beijing Key Laboratory of Assessment of Clinical Drugs Risk and Individual Application (Beijing Hospital), Beijing 100730, P.R. China

E-mail address: [xuwenfenghill@126.com](mailto:xuwenfenghill@126.com)
F-Tel: +86-18311181512

**Supplementary Methods**

**Decomposition Analysis:**

We used the standardization and decomposition methodology proposed by Das Gupta to decompose the absolute changes in DALYs, deaths, and prevalent cases for CDs from 1990 to 2023 into the contributions of three mutually exclusive factors: population growth, population age structure, and epidemiologic changes.

Taking DALYs as an example, the total number of DALYs in a specific year y is determined by the multiplicative interaction of these three factors:

DALY _ay, py, ey_ = $\sum_{\text{i=1}}^{\text{n}} \text{(}$a _i, y_ * p _y_ * e _i, y_)

Where DALY_ay, py, ey_ represented DALYs based on the factors of age structure, population, and DALYs rate for specific year y; a_i, y_ represents the proportion of population for the age category i of the n age categories in given year y; p_y_ represents the total population in given year y; and e_i, y_ represents DALYs rate given age category i in year y.

1. Rationale for the Weighting Scheme

When decomposing the difference in DALYs between 1990 (t1) and 2023 (t2), simply changing one factor while holding others constant at the baseline (t1) or endpoints (t2) would yield different results depending on the arbitrary order of decomposition. To solve this path-dependent problem and eliminate interaction effects, the Das Gupta method applies a specific weighting scheme.

The rationale is to calculate the effect of a target factor by averaging its changes across all possible combinations of the remaining factors at times t1 and t2. For a three-factor decomposition, there are 2^3-1^ = 4 possible contexts for the other two factors. The weighting scheme assigns equal weights to these contexts based on combinatorial symmetry, satisfying the factor-reversal test and ensuring that the sum of individual effects perfectly equals the total change (Δ Total = Δ a + Δ p + Δ e).

2. Derivation of the Equations

The net effect of the population age structure (Δ a) is derived by holding the other two factors (p and e) constant in all 4 possible combinations, expressed as:

Δa = [(DALY _a2023, p1990, e1990_ - DALY _a1990, p1990, e1990_)/3+ (DALY _a2023, p2023, e2023_ - DALY _a1990, p2023, e2023_)/3] + [(DALY _a2023, p1990, e2023_ - DALY _a1990, p1990, e2023_)/6+ (DALY _a2023, p2023, e1990_ - DALY _a1990, p2023, e1990_)/6].

By factoring out the common terms, the algebraic derivation simplifies to the final operational weighting equation:

Δa = [(DALY _a2023, p1990, e1990_ + DALY _a2023, p2023, e2023_)/3+ (DALY _a2023, p1990, e2023_ + DALY _a2023, p2023, e1990_)/6] - [(DALY _a1990, p2023, e2023_ + DALY _a1990, p1990, e1990_)/3+ (DALY _a1990, p2023, e1990_ + DALY _a1990, p1990, e2023_)/6].

Similarly, the effects of population growth (Δ p) and epidemiologic changes (Δ e) are symmetrically derived:

Δp = [(DALY _a1990, p2023, e1990_ + DALY _a2023, p2023, e2023_)/3+ (DALY _a1990, p2023, e2023_ + DALY _a2023, p2023, e1990_)/6] - [(DALY _a2023, p1990, e1990_ + DALY _a1990, p1990, e1990_)/3+ (DALY _a2023, p1990, e2023_ + DALY _a1990, p1990, e1990_)/6].

Δe = [(DALY _a1990, p1990, e2023_ + DALY _a2023, p2023, e2023_)/3+ (DALY _a1990, p2023, e2023_ + DALY _a2023, p1990, e2023_)/6] - [(DALY _a2023, p2023, e1990_ + DALY _a1990, p1990, e1990_)/3+ (DALY _a2023, p1990, e1990_ + DALY _a1990, p2023, e1990_)/6].

**Joinpoint regression analysis:**

We used Joinpoint regression to evaluate temporal trends in TD burden. Using a segmented log-linear regression model shown in Eq. (1), we identified trend inflection points.

$$\begin{aligned} \ln\left( y \right)=\beta\times x\#\left( 1 \right) \end{aligned}$$

Where y represents the age-standardized rate (specifically ASPR, ASMR, or ASDR) of the disease. ln(y) denotes the natural logarithm of the rate, which is utilized to stabilize variance and address potential heteroscedasticity. x denotes the calendar year (independent variable). $\beta$ represents the regression coefficient (slope) for the corresponding time segment, from which the Annual Percentage Change (APC) is derived.

Autocorrelated errors were not modeled and rates were log-transformed to stabilize variance and address potential heteroscedasticity. Model parameter optimization was performed through permutation testing employing a significance level (α) set at 0.05 and 4,499 permutations. Joinpoint significance was evaluated against this pre-specified α criterion. The number of joinpoints was specified to vary between 0 and 6, and the program determined the optimal number of joinpoints for the model. Model fit was assessed using standard diagnostic plots of log-transformed residuals to evaluate homoscedasticity, normality, and independence of errors. We conducted the analysis using Joinpoint software and calculated the APC and AAPC to quantify the temporal trends in disease burden from 1990 to 2023. The APC was calculated using Eq. (2):

$$\begin{aligned} APC=\left[ \exp\left( \beta\right)-1 \right]\times100\%\#\left( 2 \right) \end{aligned}$$

Where β is the regression coefficient derived from the log-linear model in Eq. (1). The AAPC represents the overall average trend between 1990 and 2023. It is computed as a weighted average of the APCs across all segments, with the weights assigned to each segment. The formula is shown in Eq. (3):

$$\begin{aligned} AAPC=\left[ \exp\left( \frac{\sum w_{i}b_{i}}{\sum w_{i}} \right)-1 \right]\times100\#\left( 3 \right) \end{aligned}$$

Where wᵢ denotes the length of each segment, and bᵢ is the corresponding slope coefficient derived from the log-linear model for that segment. If the 95% CI for the AAPC estimate is greater than 0, it indicates an increasing trend; if less than 0, a decreasing trend; and if equal to 0, a stable trend.

**BAPC model: prior specifications**

For Bayesian age–period–cohort (BAPC) projections, we modelled age, period, and cohort effects using a latent Gaussian age–period–cohort model with Poisson likelihood and log link. Age-, period-, and cohort-specific case counts $\text{Y}_{\text{a,p}}$ were assumed to follow a Poisson distribution, $\text{Y}_{\text{a,p}}\text{∼Poisson}\left( \text{E}_{\text{a,p}}\text{λ}_{\text{a,p}} \right)$, where $\text{E}_{\text{a,p}}$ denotes the population at risk and $\text{λ}_{\text{a,p}}$ the underlying rate. The log-rate was decomposed as $\log\text{(}\text{λ}_{\text{a,p}}\text{)=α+}\text{f}_{\text{age}}\text{(a)+}\text{f}_{\text{period}}\text{(p)+}\text{f}_{\text{cohort}}\text{(c)+}\text{u}_{\text{a,p}}$. The three temporal components $\text{f}_{\text{age}}\text{,}\text{f}_{\text{period}}\text{,}\text{f}_{\text{cohort}}$ were assigned second-order random walk (RW2) priors. For the corresponding precision parameters ($\text{τ}_{\text{age}}\text{,}\text{τ}_{\text{period}}\text{,}\text{τ}_{\text{cohort}}$), we used the default weakly informative log-Gamma priors implemented in BAPC/INLA, $\log\left( \text{τ}_{\text{k}} \right)\text{∼logGamma}\left( \text{1, 0.00005} \right)\text{, k∈\{age,period,cohort\}}$. An additional overdispersion term $\text{u}_{\text{a,p}}$ was included and modelled as iid $\text{N}\left( \text{0,}\text{τ}_{\text{overdis}}^{\text{-1}} \right)$ with $\text{ }\log\left( \text{τ}_{\text{overdis}} \right)\text{∼logGamma}\left( \text{1,0.005} \right)$. Models were fitted via the BAPC package (version 0.0.36) using Integrated Nested Laplace Approximations (INLA).

**Supplementary Figure**

Supplementary Figure S1 Joinpoint regression analysis of the sex-specific trends in ASPR (per 100,000 population), ASMR (per 100,000 population), and ASDR (per 100,000 population) for Crohn's disease from 1990 to 2023 in Republic of Korea.

ASPR: age-standardized prevalence rate; ASMR: age-standardized mortality rate; ASDR: age-standardized disability-adjusted life years rate.

Supplementary Figure S2 Joinpoint regression analysis of the sex-specific trends in ASPR (per 100,000 population), ASMR (per 100,000 population), and ASDR (per 100,000 population) for Crohn's disease from 1990 to 2023 in Japan.

ASPR: age-standardized prevalence rate; ASMR: age-standardized mortality rate; ASDR: age-standardized disability-adjusted life years rate.

Supplementary Figure S3 Joinpoint regression analysis of the sex-specific trends in ASPR (per 100,000 population), ASMR (per 100,000 population), and ASDR (per 100,000 population) for Crohn's disease from 1990 to 2023 in China.

ASPR: age-standardized prevalence rate; ASMR: age-standardized mortality rate; ASDR: age-standardized disability-adjusted life years rate.

Supplementary Figure S4 Age-specific numbers and rates (per 100,000 population) of mortality rate number due to CD by gender in 2023. (A) China; (B) Japan; (C) Republic of Korea.

CD: Crohn’s Disease; DALYs: disability-adjusted life years.

**Supplementary Table**

Supplementary Table S1 Decomposition analysis of CD burden in China, Japan, and the Republic of Korea

| location | sex | cause | measure | Overll difference | Aging | Population | Epidemiological change |
| --- | --- | --- | --- | --- | --- | --- | --- |
| Republic of Korea | Male | Crohn's disease | Deaths | 1.68(14.5%) | 4.9  (42.35%) | 4.28  (37.03%) | -7.51  (-64.88%) |
| Republic of Korea | Female | Crohn's disease | Deaths | -2.8(-23.15%) | 8.13  (67.28%) | 4.01  (33.18%) | -14.94  (-123.61%) |
| Republic of Korea | Both | Crohn's disease | Deaths | -1.12(-4.74%) | 13.43  (56.76%) | 8.36  (35.35%) | -22.91  (-96.85%) |
| Republic of Korea | Male | Crohn's disease | DALYs (Disability-Adjusted Life Years) | 462.47(35.59%) | 78.77  (6.06%) | 509.56  (39.21%) | -125.85  (-9.68%) |
| Republic of Korea | Female | Crohn's disease | DALYs (Disability-Adjusted Life Years) | 154.93(14.93%) | 122.12  (11.77%) | 363.27  (35%) | -330.46  (-31.84%) |
| Republic of Korea | Both | Crohn's disease | DALYs (Disability-Adjusted Life Years) | 617.4(26.41%) | 194.04  (8.3%) | 870.84  (37.26%) | -447.48  (-19.14%) |
| Republic of Korea | Male | Crohn's disease | Prevalence | 3977.06(86.79%) | 303.65  (6.63%) | 2128.85  (46.46%) | 1544.56  (33.71%) |
| Republic of Korea | Female | Crohn's disease | Prevalence | 2891.56(88.8%) | 326.12  (10.01%) | 1450.22  (44.53%) | 1115.23  (34.25%) |
| Republic of Korea | Both | Crohn's disease | Prevalence | 6868.63(87.62%) | 560.43  (7.15%) | 3564.08  (45.47%) | 2744.13  (35.01%) |
| Japan | Male | Crohn's disease | Deaths | 30.03(74.73%) | 36.25  (90.2%) | 4.41  (10.97%) | -10.62  (-26.44%) |
| Japan | Female | Crohn's disease | Deaths | -5.27(-13.84%) | 45.05  (118.35%) | 4.16  (10.93%) | -54.48  (-143.13%) |
| Japan | Both | Crohn's disease | Deaths | 24.76(31.64%) | 80  (102.24%) | 8.71  (11.13%) | -63.95  (-81.72%) |
| Japan | Male | Crohn's disease | DALYs (Disability-Adjusted Life Years) | 603.36(32.53%) | 380.88  (20.54%) | 166.35  (8.97%) | 56.13  (3.03%) |
| Japan | Female | Crohn's disease | DALYs (Disability-Adjusted Life Years) | 46.22(2.93%) | 458.65  (29.11%) | 156.37  (9.93%) | -568.8  (-36.1%) |
| Japan | Both | Crohn's disease | DALYs (Disability-Adjusted Life Years) | 649.59(18.94%) | 812.91  (23.7%) | 327.81  (9.56%) | -491.13  (-14.32%) |
| Japan | Male | Crohn's disease | Prevalence | 1753.84(52.42%) | 214.27  (6.4%) | 320.4  (9.58%) | 1219.18  (36.44%) |
| Japan | Female | Crohn's disease | Prevalence | 2361.58(58.06%) | 225.71  (5.55%) | 480.06  (11.8%) | 1655.81  (40.71%) |
| Japan | Both | Crohn's disease | Prevalence | 4115.43(55.51%) | 470.9  (6.35%) | 794.79  (10.72%) | 2849.74  (38.44%) |
| China | Male | Crohn's disease | Deaths | 68.56(110.5%) | 40.56  (65.37%) | 27.68  (44.62%) | 0.31  (0.51%) |
| China | Female | Crohn's disease | Deaths | -15.22(-17.57%) | 56.76  (65.53%) | 29.63  (34.21%) | -101.61  (-117.31%) |
| China | Both | Crohn's disease | Deaths | 53.34(35.88%) | 99.73  (67.08%) | 57.59  (38.74%) | -103.97  (-69.94%) |
| China | Male | Crohn's disease | DALYs (Disability-Adjusted Life Years) | 4145.5(96.1%) | 707.69  (16.41%) | 1845.78  (42.79%) | 1592.02  (36.91%) |
| China | Female | Crohn's disease | DALYs (Disability-Adjusted Life Years) | 2203.98(42.85%) | 1105.14  (21.49%) | 2031.95  (39.51%) | -933.1  (-18.14%) |
| China | Both | Crohn's disease | DALYs (Disability-Adjusted Life Years) | 6349.48(67.14%) | 1822.18  (19.27%) | 3875.79  (40.99%) | 651.51  (6.89%) |
| China | Male | Crohn's disease | Prevalence | 18371.9(189.69%) | 1841.04  (19.01%) | 5301.4  (54.74%) | 11229.46  (115.95%) |
| China | Female | Crohn's disease | Prevalence | 22194.68(195.84%) | 2391.93  (21.11%) | 6735.82  (59.44%) | 13066.93  (115.3%) |
| China | Both | Crohn's disease | Prevalence | 40566.58(193.01%) | 4270.36  (20.32%) | 11992.49  (57.06%) | 24303.74  (115.63%) |

Supplementary Table S2 Model diagnostics for BAPC

| Outcome | Location | Sex | DIC | Effective number of parameters | CPO failures | PIT mean | PIT median |
| --- | --- | --- | --- | --- | --- | --- | --- |
| Prevalence | China | Male | 6294.38 | 442.86 | 0 | 0.478 | 0.499 |
| Prevalence | China | Female | 6518.21 | 473.89 | 0 | 0.467 | 0.483 |
| Prevalence | Japan | Male | 4770.09 | 95.89 | 0 | 0.539 | 0.554 |
| Prevalence | Japan | Female | 5078.2 | 111.58 | 0 | 0.531 | 0.539 |
| Prevalence | Republic of Korea | Male | 5251.57 | 434.19 | 0 | 0.478 | 0.501 |
| Prevalence | Republic of Korea | Female | 5242.12 | 431.88 | 0 | 0.469 | 0.508 |
| DALYs | China | Male | 5513.886 | 134.27 | 0 | 0.518 | 0.553 |
| DALYs | China | Female | 6016.45 | 319.62 | 0 | 0.503 | 0.523 |
| DALYs | Japan | Male | 4296.24 | 68.05 | 0 | 0.53 | 0.544 |
| DALYs | Japan | Female | 4340.65 | 71.01 | 0 | 0.528 | 0.535 |
| DALYs | Republic of Korea | Male | 4134.28 | 149.36 | 0 | 0.557 | 0.603 |
| DALYs | Republic of Korea | Female | 4227.99 | 118.03 | 0 | 0.536 | 0.567 |

DIC, Deviance Information Criterion; CPO, Conditional Predictive Ordinate; PIT, Probability Integral Transform; DALYs, disability-adjusted life years.

Supplementary Table S3 Sensitivity analyses of BAPC projection of prevalence cases and DALYs

| Year | China | | Japan | | Republic of Korea | |
| --- | --- | --- | --- | --- | --- | --- |
|  | DALYs | Prevalence | DALYs | Prevalence | DALYs | Prevalence |
| 1990 | 11030.85 | 21581.62 | 3469.03 | 7557.41 | 2433.95 | 7973.37 |
| 1991 | 11395.24 | 22996.11 | 3531.54 | 8780.76 | 2449.18 | 8256.96 |
| 1992 | 11766.87 | 24613.84 | 3611.48 | 9965.48 | 2441.73 | 8504.13 |
| 1993 | 12168.00 | 26437.88 | 3687.17 | 10963.88 | 2447.29 | 8747.71 |
| 1994 | 12588.34 | 28436.90 | 3740.43 | 11674.11 | 2452.47 | 8977.58 |
| 1995 | 13033.28 | 30583.44 | 3751.19 | 12039.96 | 2454.89 | 9193.74 |
| 1996 | 13596.08 | 33374.29 | 3716.00 | 12088.79 | 2457.21 | 9398.17 |
| 1997 | 14283.19 | 36919.51 | 3667.43 | 11958.19 | 2448.42 | 9582.41 |
| 1998 | 15042.78 | 40605.03 | 3627.90 | 11775.08 | 2449.92 | 9776.65 |
| 1999 | 15784.61 | 43724.87 | 3611.69 | 11642.76 | 2457.56 | 10012.68 |
| 2000 | 16400.79 | 45537.60 | 3612.33 | 11709.82 | 2480.40 | 10311.07 |
| 2001 | 16726.90 | 46018.27 | 3666.63 | 12214.83 | 2532.43 | 10822.93 |
| 2002 | 16916.92 | 45881.33 | 3787.51 | 13191.87 | 2615.85 | 11583.46 |
| 2003 | 16964.99 | 45564.37 | 3951.99 | 14377.74 | 2735.83 | 12481.52 |
| 2004 | 16962.93 | 45466.39 | 4073.64 | 15390.40 | 2845.82 | 13377.93 |
| 2005 | 16662.83 | 46024.26 | 4143.22 | 15830.28 | 2949.94 | 14138.68 |
| 2006 | 16628.06 | 49334.31 | 4101.13 | 15452.40 | 3060.67 | 14891.81 |
| 2007 | 17189.16 | 55935.04 | 3983.25 | 14452.31 | 3196.67 | 15754.84 |
| 2008 | 18014.01 | 63809.59 | 3833.25 | 13252.22 | 3302.30 | 16549.99 |
| 2009 | 18887.34 | 70770.10 | 3680.52 | 12265.12 | 3385.83 | 17132.00 |
| 2010 | 19313.33 | 74599.43 | 3573.58 | 11776.53 | 3412.24 | 17359.60 |
| 2011 | 19274.15 | 75689.37 | 3517.52 | 11678.17 | 3358.11 | 17107.52 |
| 2012 | 19045.49 | 76081.34 | 3493.70 | 11682.72 | 3258.61 | 16469.58 |
| 2013 | 18761.50 | 75958.12 | 3483.93 | 11707.27 | 3152.66 | 15655.36 |
| 2014 | 18547.15 | 75436.22 | 3496.43 | 11723.78 | 3036.61 | 14895.90 |
| 2015 | 18365.28 | 74649.83 | 3536.65 | 11726.41 | 2953.92 | 14413.44 |
| 2016 | 18023.86 | 72360.54 | 3595.66 | 11709.89 | 2906.64 | 14220.29 |
| 2017 | 17440.52 | 68235.93 | 3683.05 | 11676.50 | 2895.34 | 14176.56 |
| 2018 | 16823.01 | 63718.16 | 3740.36 | 11637.21 | 2907.45 | 14259.47 |
| 2019 | 16365.36 | 60185.19 | 3776.33 | 11601.68 | 2926.16 | 14403.93 |
| 2020 | 16222.19 | 58917.27 | 3841.01 | 11588.28 | 2940.19 | 14560.24 |
| 2021 | 16411.43 | 60067.43 | 3922.62 | 11610.60 | 2951.95 | 14698.46 |
| 2022 | 16623.21 | 61491.18 | 4012.40 | 11627.61 | 2953.38 | 14779.81 |
| 2023 | 16384.51 | 62302.93 | 4100.73 | 11583.93 | 2970.14 | 14795.04 |
| 2024 | 16901.99 | 67389.54 | 4202.66 | 11567.99 | 3001.93 | 15541.26 |
| 2025 | 16892.04 | 68913.46 | 4304.09 | 11549.62 | 3009.19 | 15619.76 |
| 2026 | 16886.71 | 70600.89 | 4413.38 | 11553.03 | 3017.00 | 15699.64 |
| 2027 | 16895.16 | 72496.13 | 4532.10 | 11585.65 | 3025.49 | 15784.08 |
| 2028 | 16941.71 | 74651.65 | 4662.07 | 11663.40 | 3036.02 | 15873.06 |
| 2029 | 17032.36 | 77167.81 | 4804.29 | 11797.45 | 3049.28 | 15983.90 |
| 2030 | 17126.10 | 80164.16 | 4959.74 | 12002.87 | 3066.02 | 16119.18 |
| 2031 | 17232.08 | 83695.56 | 5133.02 | 12291.15 | 3088.69 | 16289.40 |
| 2032 | 17356.17 | 87891.15 | 5331.19 | 12679.30 | 3118.47 | 16503.75 |
| 2033 | 17531.79 | 92923.94 | 5556.30 | 13190.73 | 3156.82 | 16766.82 |
| 2034 | 17772.12 | 98967.16 | 5810.75 | 13855.65 | 3205.24 | 17093.20 |
| 2035 | 18043.13 | 106326.97 | 6097.74 | 14706.91 | 3265.87 | 17496.09 |
| 2036 | 18353.96 | 115223.07 | 6428.41 | 15794.09 | 3342.10 | 17994.99 |
| 2037 | 18716.61 | 126099.56 | 6814.89 | 17190.57 | 3437.17 | 18620.38 |
| 2038 | 19160.03 | 139530.35 | 7267.22 | 18987.37 | 3554.63 | 19394.41 |
| 2039 | 19694.32 | 156245.61 | 7804.21 | 21315.18 | 3698.04 | 20351.01 |
| 2040 | 20299.27 | 177369.69 | 8444.81 | 24363.23 | 3874.44 | 21540.11 |
| 2041 | 20982.33 | 204176.95 | 9228.47 | 28407.73 | 4092.83 | 23031.39 |
| 2042 | 21761.46 | 238747.11 | 10210.74 | 33846.95 | 4363.50 | 24921.76 |
| 2043 | 22697.87 | 284008.52 | 11465.18 | 41320.23 | 4700.90 | 27333.00 |
| 2044 | 23812.25 | 344219.08 | 13098.35 | 51813.31 | 5123.10 | 30441.50 |
| 2045 | 25092.13 | 425960.19 | 15267.92 | 66914.92 | 5656.30 | 34505.93 |
| 2046 | 26575.63 | 538672.61 | 18232.75 | 89217.56 | 6339.73 | 39901.04 |
| 2047 | 28307.21 | 697367.54 | 22378.38 | 123080.67 | 7225.59 | 47193.55 |
| 2048 | 30438.74 | 925648.27 | 28372.82 | 176012.33 | 8387.95 | 57198.49 |
| 2049 | 33001.73 | 1261580.36 | 37266.16 | 261327.68 | 9939.85 | 71213.17 |
| 2050 | 36031.45 | 1767437.48 | 50830.12 | 403075.08 | 12044.79 | 91222.84 |
| 2051 | 39653.88 | 2546542.77 | 72106.43 | 645813.53 | 14949.40 | 120345.92 |
| 2052 | 44013.49 | 3773312.53 | 106190.00 | 1073867.30 | 19029.26 | 163602.96 |
| 2053 | 49459.64 | 5751521.69 | 162644.26 | 1851098.44 | 24865.30 | 229105.65 |
| 2054 | 56217.04 | 9017251.53 | 258129.22 | 3301521.82 | 33357.23 | 330321.19 |
| 2055 | 64488.90 | 14520282.21 | 423265.08 | 6080708.83 | 45929.56 | 489630.86 |
| 2056 | 74840.16 | 23997623.19 | 715042.53 | 11541390.04 | 64859.44 | 744752.10 |
| 2057 | 87854.24 | 40643442.87 | 1239436.47 | 22507564.03 | 93847.38 | 1160630.82 |
| 2058 | 104973.09 | 70496010.48 | 2203782.13 | 45064843.70 | 138930.85 | 1849393.37 |
| 2059 | 127499.88 | 125085500.11 | 4005617.07 | 92398805.46 | 210072.12 | 3008731.95 |
| 2060 | 156444.43 | 226496612.55 | 7426469.49 | 193693936.69 | 324001.40 | 4989162.52 |
